# Masking of iron and other interferences during the spectrophotometric analysis of aluminum from soil samples using Alizarin Red S

**DOI:** 10.55730/1300-0527.3713

**Published:** 2024-09-17

**Authors:** Hafiz Zain Ul AABIDIN, Muhammad Inam Ul HASSAN, Tariq YASIN, Muhammad Zubair RAHIM, Ahsan Amin BHATTI, Hamayun IMTIAZ, Asif RAZA

**Affiliations:** Department of Chemical Engineering, Pakistan Institute of Engineering and Applied Sciences, Nilore, Islamabad, Pakistan

**Keywords:** Masking of iron, titanium, spectrophotometric, analysis of aluminum, Alizarin Red S, geological samples

## Abstract

In whole-rock geochemical analysis, the quantification of aluminum in geological samples is performed using different analytical techniques including UV-Vis spectrophotometry. During the spectrophotometric analysis of aluminum utilizing Alizarin Red S, iron causes significant interference, leading to inaccurate measurements of the amount of aluminum in the sample. In cases where leached or processed samples contain titanium, interference is also observed. However, appropriate masking agents can remove these interferences. In this study, various masking agents including Tiron, oxalic acid, ascorbic acid, and thioglycolic acid were utilized for the masking of iron. Among these masking agents, ascorbic acid proved to be the best candidate for masking iron. A simple and efficient method was then developed for the masking of iron with the optimization of parameters including concentration of ascorbic acid and reaction time. With this technique, 2 mL of ascorbic acid (10%) can mask iron at up to 3000 ppm in aliquots. Moreover, titanium interference is not observed with this method when the titanium concentration in the aliquot is below 100 ppm. This method was subsequently applied to geological samples and the results were compared to those of atomic absorption spectroscopy. Most samples can easily be analyzed for aluminum by utilizing this masking technique and circumventing interference problems.

## 1. Introduction

**Figure 1:**
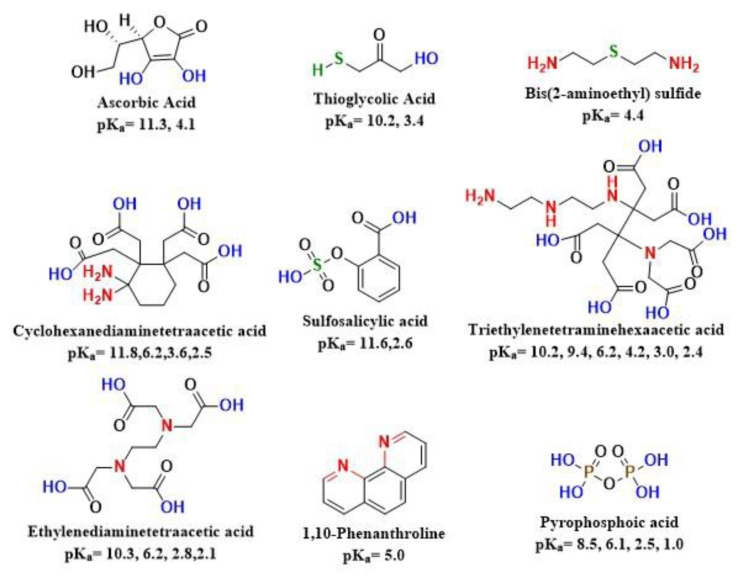
Masking agents commonly used for the masking of different elements.

The determination of aluminum in whole-rock geochemical analysis is crucial for classifying rocks and understanding the earth’s geochemical processes [1,2]. Aluminum is a key element in the earth’s crust, providing insights into mineralogical changes and rock weathering over geological timescales [3]. Its distribution in soils and sediments indicates environmental changes and human impacts, especially in areas affected by industrial pollution and mining. Additionally, aluminum’s presence in minerals such as clays and feldspars is important for mineral exploration and the assessment of economic deposits [4,5]. Therefore, the determination of aluminum is important [6]. While advanced analytical techniques such as inductively coupled plasma-mass spectrometry (ICP-MS) and atomic absorption spectroscopy (AAS) enable the precise measurement of aluminum concentrations, the importance of wet chemical procedures such as volumetry or spectrophotometry cannot be overlooked as these procedures are accessible, economical, and commonly employed for the validation of results [7,8]. However, these spectrophotometric methods are disrupted when metals in geological samples other than the analyte of interest interfere with the readings. These metals can react with the dyes used, creating colored complexes that disrupt the accurate measurement of the target analyte. This interference consequently leads to inaccurate results [9]. Addressing this challenge requires methods that are less prone to such interference or the use of suitable masking agents to reduce such interference. With advancements in complexometric analysis, different masking agents including hydroxylamine HCl, thioglycolic acid, and ethylenediaminetetraacetic acid (EDTA) were also utilized for the masking of iron but none of them gave fully promising results [10,11]. A list of important masking agents is presented in Figure 1 [12]. Typically, these masking agents have been employed in conjunction with aluminon or Alizarin Red S (ARS), which are complexing agents for aluminum [13,14].

The objective of the present study is to develop a simple, efficient, and reproducible method devoid of interference for the analysis of aluminum. Among the four masking agents of Tiron, oxalic acid, ascorbic acid, and thioglycolic acid, ascorbic acid emerged as a promising candidate. Thus, we aimed to employ ascorbic acid while optimizing different parameters for the masking of iron and titanium. The method was applied to geological samples collected from various regions of Pakistan including Gilgit-Baltistan, Azad Kashmir, and Mianwali/Punjab and the obtained results were compared with those obtained from AAS. This method utilizes ecofriendly reagents for the spectrophotometric determination of aluminum from soil and leached samples using ARS.

### 1.1. Interfering factors in aluminum analysis

Interference in the spectrophotometric estimation of aluminum arises from the chemical similarity and complexation behaviors of interfering species such as iron and titanium. These metals share the ability to form complexes with the same reagents as aluminum, leading to overlapping absorption spectra at specific wavelengths, which can result in inaccurate measurements [15,16]. Iron has a particularly significant effect on aluminum estimation, while titanium’s interference, though marginal, can further complicate the analysis. This spectral overlap makes it difficult to differentiate aluminum from these metals [17].

On the other hand, trace elements like zinc, lead, and cadmium are typically present in much smaller quantities relative to aluminum; thus, they do not usually contribute significantly to the interference. The low concentrations of these trace metals result in minimal or negligible overlap in absorption spectra, allowing for relatively clear and accurate estimation of aluminum. However, in complex geological matrices, the combined presence of multiple interfering species can make the spectrophotometric determination of aluminum more difficult, requiring advanced techniques or reagent optimization to minimize errors [18,19].

## 2. Experimental

### 2.1. Chemicals and reagents

In this study, all chemicals and reagents were of analytical grade and used without further purification unless otherwise noted. CaCl_2_ (≥99% purity), HCl (37% purity), oxalic acid (≥99.50% purity), thioglycolic acid (≥99.80% purity), ascorbic acid (≥99% purity), acetic acid (≥99.70% purity), sodium acetate (≥99% purity), ferrous ammonium sulfate (≥99% purity), ARS (≥50% dye content), and H_2_O_2_ (50% purity) were obtained from Sigma-Aldrich (St. Louis, MO, USA) and ferric chloride (99.99% purity), titanium chloride (99.99% purity), and aluminum chloride (99.99% purity) were obtained from BDH (Radnor, PA, USA). Type II water was used throughout the experiments.

### 2.2. Instrumentation

An AAS device (Z-8000, Hitachi, Tokyo, Japan) and single-beam UV-Vis spectrophotometer (PG Instruments, Lutterworth, UK) were used for analysis of aluminum, iron, and titanium. The AAS conditions were adjusted as follows for the analysis of iron: wavelength of 372.4 nm, slit value of 0.4 nm, lamp current of 10 mA, and air pressure of 1.6 kg/cm^2^. Samples were analyzed in acetylene–air flame.

For the analysis of aluminum by AAS, the slit width was set to 1.3 nm and the wavelength was adjusted to 309.3 nm. The lamp current was maintained at 10 mA and air pressure was set to 1.6 kg/cm^2^. The samples were analyzed in nitrous oxide–acetylene flame. Potassium chloride (KCl) was added as a buffer to all standards and samples to eliminate flame interference.

For the analysis of aluminum and titanium using a UV-Vis spectrophotometer, the instrument was warmed up for approximately 1 h. Readings were performed at 275 nm for aluminum and 410 nm for titanium.

### 2.3. Preparation of reagents and standard solutions

Reagents and standards were prepared as described below.

#### 2.3.1. 0.14 M CaCl_2_ solution

CaCl_2_ was weighed to 7 g and added to a 250-mL beaker in which 100 mL of type II water was added. Subsequently, 15 mL of concentrated HCl was dissolved in that mixture and the beaker was heated until the solution became clear. The solution was then cooled to room temperature and diluted to 500 mL with type II water.

#### 2.3.2. Solutions of masking agents

A 4% solution of oxalic acid, thioglycolic acid, ascorbic acid, and 2% solution of Tiron were respectively prepared. For this purpose, 4 g, 2 g, 4 mL, and 4 g of oxalic acid, Tiron, thioglycolic acid, and ascorbic acid were respectively added to separate volumetric flasks of 100 mL. The total volume of 100 mL was achieved with type II water.

#### 2.3.3. 10% solution of ascorbic acid

A 10% solution of ascorbic acid was prepared using Eq. (1). For this purpose, 10 g of ascorbic acid was added to a 100-mL volumetric flask and the total volume was made up to 100 mL with type II water. This solution can be used for 1 week. Subsequently, the browning of ascorbic acid begins due to self-polymerization.


(1) $$\text{Percent solution }(\%)=\frac{\text{Grams of ascorbic acid}}{100\;\text{mL of solution}}\times 100$$

#### 2.3.4. Buffer solution (CH_3_COOH/CH_3_COONa)

Sodium acetate (CH_3_COONa·3H_2_O, 100 g) was added to a 500-mL beaker together with 200 mL of type II water and 30 mL of glacial acetic acid. After thorough mixing, the total volume of the solution was made up to 500 mL. The pH range of this solution was 3.6 to 5.6.

#### 2.3.5. 0.05% solution of ARS

ARS was weighed to 0.5 g and added to a 1-L beaker. The total volume was made up to 1000 mL with type II water.

#### 2.3.6. Ferric chloride solution

Ferrous ammonium sulfate [(NH_4_)_2_Fe(SO_4_)_2_.6H_2_O] was used for the preparation of standard solutions of ferric chloride. For preparation of the standard stock solution of ferric chloride (5000 ppm), 17.19 g of [(NH_4_)_2_Fe(SO_4_)_2_.6H_2_O] was added to a 500-mL beaker together with 100 mL of type II water, 26 mL of HCl (37%), and 2 mL of 50% H_2_O_2_. The beaker was covered with watch glass, heated for 5 min at 70 °C, and cooled to room temperature. Finally, the solution was transferred to a 500-mL volumetric flask and the total volume of 500 mL was achieved with type II water [20].

#### 2.3.7. Aluminum and ferric ion samples

Eight standard samples of known concentrations, containing constant concentrations of aluminum (50 ppm) but variable concentrations of iron (0, 50, 100, 500, 1000, 2000, 3000, and 4000 ppm), were prepared by taking 2.5 mL of standard aluminum solution from the 1000 ppm stock solution and 0, 5, 10, 20, 30, or 40 mL of standard iron solution from the 5000 ppm stock solution, respectively. The total volume was made up to 50 mL with type II water.

#### 2.3.8. Aluminum and titanium samples

Six standard samples with known concentrations of aluminum and titanium, containing constant concentrations of aluminum (50 ppm) but variable concentrations of titanium (25, 50, 75, 100, 150, and 200 ppm), were prepared by taking 2.5 mL of standard aluminum solution from the 1000 ppm stock solution and 1.2, 2.5, 3.75, 5, 7.5, or 10 mL of standard titanium solution from the 1000 ppm stock solution, respectively. The total volume was made up to 50 mL with type II water.

### 2.4. Optimization of parameters for the masking of iron

#### 2.4.1. Selection of masking agent

In the spectrophotometric analysis of aluminum, four different masking agents were evaluated to identify the most effective among them. In this process, 2 mL of 4% oxalic acid, 2 mL of 2% Tiron, 2 mL of 4% ascorbic acid, and 2 mL of 4% thioglycolic acid were used separately as masking agents. Six volumetric flasks were rinsed with distilled water. One flask was used for the blank; four flasks were used for the preparation of 25, 50, 75, and 100 ppm standard solutions of Al; and the remaining flask contained a standard sample (50 ppm Al and 100 ppm Fe). Subsequently, 2 mL of CaCl_2_ solution and 2 mL of 4% acid were added to all flasks. Flasks were mixed thoroughly and left for 5 min. After that, 10 mL of buffer solution and 10 mL of ARS were added. Finally, the total volume was achieved with type II water and readings were performed after 2 h at 475 nm with a spectrophotometer. The amount of aluminum in the samples was calculated with Eq. (2):


$$\text{Concentration of Al }(\text{ppm})=\frac{\text{X}}{\text{V}1}\times \text{DF}$$

Here, X is the amount of Al obtained from the calibration curve (μg), V1 is the volume of sample taken (mL), and DF is the dilution factor.

The same procedure was repeated with different types of masking agents (Tiron, ascorbic acid, and thioglycolic acid). With the procedure described above, Tiron, ascorbic acid, and thioglycolic acid were used separately in place of oxalic acid.

#### 2.4.2. Variation of ascorbic acid and iron concentrations

The concentration of ascorbic acid as a masking agent was varied as 1%, 3%, 5%, 7%, and 10%. For 1% ascorbic acid, 13 graduated flasks of 100 mL were rinsed thoroughly with distilled water. One of these flasks was used for the blank (containing only reagents); four flasks were used for the preparation of 25, 50, 75, and 100 ppm standard solutions of Al, from which the calibration curve was formed; and 1 mL of aliquot (standard samples having constant concentrations of aluminum but variable concentrations of iron) was pipetted into each of the remaining eight flasks. For the determination of aluminum, 2 mL of CaCl_2_ solution and 2 mL of 1% ascorbic acid were added to all flasks and mixed for 5 min. After that, 10 mL of buffer solution and 10 mL of ARS were added. Finally, the total volume was achieved with type II water and readings were performed after 2 h at 475 nm with a spectrophotometer. The amount of aluminum in samples was calculated with Eq. (2). The same procedure was repeated with different concentrations of ascorbic acid (3%, 5%, 7%, and 10%).

#### 2.4.3. Maximum masking limit of 10% ascorbic acid for iron

Six samples were prepared in which the concentration of aluminum was kept constant (50 ppm) and the concentration of iron varied as 3000, 3200, 3400, 3600, 3800, and 4000 ppm. Eleven volumetric flasks of 100 mL were rinsed thoroughly with distilled water. One of these flasks was used for the blank (containing only reagents); four flasks were used for the preparation of 25, 50, 75, and 100 ppm standard solutions of Al, from which the calibration curve was formed; and 1 mL of aliquot (standard samples having variable concentrations of iron) was pipetted separately into each of the remaining six flasks. For the determination of aluminum, the method described in Section 2.4.2 was used.

### 2.5. Variation in concentration of Ti for masking

The samples prepared as described in Section 2.3.8 were used to study the effect of Ti concentration. Eleven volumetric flasks of 100 mL were rinsed thoroughly with distilled water. One of these flasks was used for the blank (containing only reagents); four flasks were used for the preparation of 25, 50, 75, and 100 ppm standard solutions of Al, from which the calibration curve was formed; and 1 mL of aliquot (standard samples having variable concentrations of titanium) from six samples was pipetted separately into each of the remaining six flasks. The quantity of aluminum was determined as described in Section 2.4.2.

### 2.6. Sample fusion

A mixture of potassium carbonate, sodium carbonate, and boric acid (2.5 g, 1:1:1) and 0.2 g of ground soil or clay were combined in a platinum crucible and heated to about 380 °C on a Bunsen burner for 40 min or until a clear solution was obtained. The crucible was then placed in a 100-mL beaker containing 30 mL of distilled water. Subsequently, 10 mL of 1:1 nitric acid was added to the beaker and the crucible was removed. The total volume was made up to 100 mL with distilled water [21].

### 2.7. Procedure for analysis of Al using UV-Vis spectrophotometry

For the preparation of 25, 50, 75, and 100 ppm standard solutions, 0.25, 0.5, 0.75, or 1 mL of standard aluminum solution was respectively taken from the 100 ppm stock solution and pipetted into a 100-mL volumetric flask. An appropriate volume of fused sample was pipetted into a separate flask. Reagents were then added to all flasks in the following order: 2 mL of 0.14 M CaCl_2_, followed by 2 mL of 10% ascorbic acid. The contents were mixed for 5 min. After that, 10 mL of buffer solution was added to all flasks followed by mixing together with the addition of 10 mL of 0.05% ARS. Finally, the total volume was made up to 100 mL with type II water and readings were performed after 100 min at 475 nm with a spectrophotometer. The amount of aluminum was calculated from solid samples using Eq. (3):


(2) $$\text{Concentration of Al }(\text{ppm})=\frac{\text{X}\times \text{W}}{\text{V}1\times \text{V}}\times \text{DF}$$

Here, X is the amount of Al obtained from the calibration curve (μg), W is the weight of the sample taken for fusion (g), V1 is the volume of the sample taken (mL), V is the total volume of the solution made after fusion of the sample (mL), and DF is the dilution factor. W and V were not used for the liquid samples as no fusion is required for liquid samples.

### 2.8. Analysis of iron by AAS

Samples were prepared according to the procedure described in Section 2.6 or direct samples were used in the case of liquids. Standard iron solutions with concentrations of 15, 30, 50, or 100 ppm were prepared from a 1000 ppm stock solution using the dilution method. After adjusting the instrumental conditions as described in Section 2.2 for iron, the blank, standards, and samples were aspirated in flame and readings were performed. The factor (F) value was obtained from the calibration curve formed by standard solutions. The concentration of iron in the samples was calculated with Eq. (4):


(3) $$\text{Concentration of Fe }(\text{ppm})=\text{X}\times \text{DF}\times \frac{Value\;of\;std\;calibration\;curve}{Known\;value\;of\;std\;solution}$$

Here, X is the amount of Fe obtained from the instrument (μg), DF is the dilution factor, and std is the standard [22].

### 2.9. Analysis of aluminum by AAS

For the analysis of aluminum, samples were prepared following the method described in Section 2.6 or direct samples were used in the case of liquids. Standard aluminum solutions with concentrations of 15, 30, 50, and 100 ppm were prepared from a 1000 ppm stock solution using the dilution formula. The instrumental conditions were adjusted as outlined in Section 2.2. Samples were aspirated into the flame and readings were performed carefully. The factor (F) value was determined from the calibration curve established using standard solutions [23]. Consequently, the experimental concentration of aluminum in the samples was determined using Eq. (4).

## 3. Results and discussion

### 3.1. Modification of the method

#### 3.1.1. Selection of masking agent

**Figure 2:**
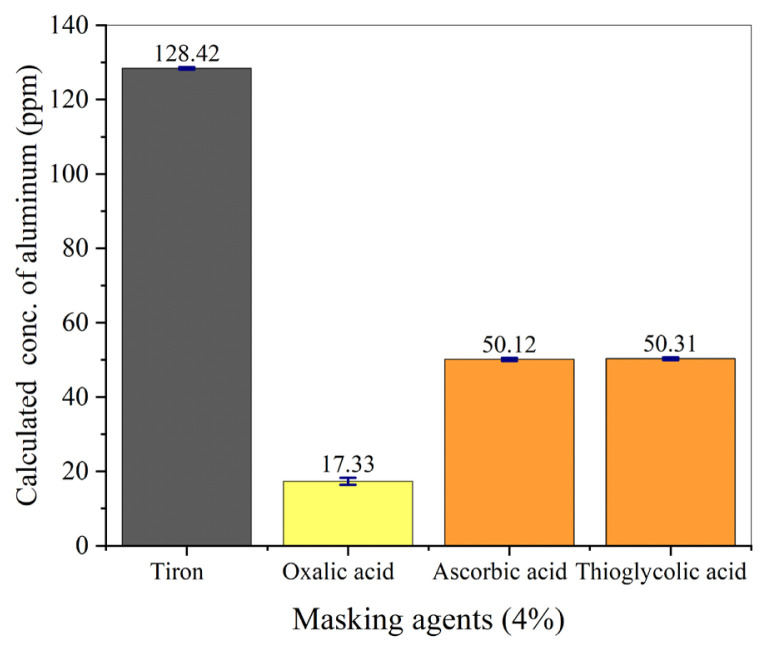
Effect of iron and various masking agents on calculated concentration of aluminum.

The selection of a masking agent is critical because a good masking agent removes interferences without reacting with the analyte of interest. In spectrophotometric analysis of aluminum from geological samples, iron was masked by applying various masking agents including Tiron, oxalic acid, ascorbic acid, and thioglycolic acid. The impact of these ligands was evaluated by examining their effects on known concentrations of aluminum (50 ppm) and iron (100 ppm) [24]. Figure 2 indicates that the different masking agents affected the masking of iron and the calculated concentration of aluminum in different ways. The concentration of aluminum was measured as 128.42, 17.33, 50.12, and 50.31 ppm in experiments in which Tiron, oxalic acid, ascorbic acid, and thioglycolic acid were used for masking, respectively. It was found that the calculated concentration of aluminum was higher than its actual concentration measured experimental when using Tiron as the masking agent due to the formation of the gray-colored complex of Tiron with iron, which exhibited a maximum absorption peak at 475 nm [25,26]. Conversely, when oxalic acid served as the masking agent, the calculated aluminum concentration was lower than the actual concentration due to the formation of the yellow-colored complex of oxalic acid with the oxides and nitrates of both aluminum and iron [27]. Therefore, these complexes did not exhibit an absorption peak at 475 nm. In the experiments utilizing thioglycolic acid and ascorbic acid as masking agents, no disparity was noted between the actual and experimental concentrations of aluminum, as the complexes with iron did not exhibit an absorption peak around 475 nm. Of the two, thioglycolic acid was disregarded as it poses serious health hazards, leading to the selection of ascorbic acid as the preferred masking agent for iron.

#### 3.1.2. Variation of ascorbic acid and iron concentrations

**Figure 3:**
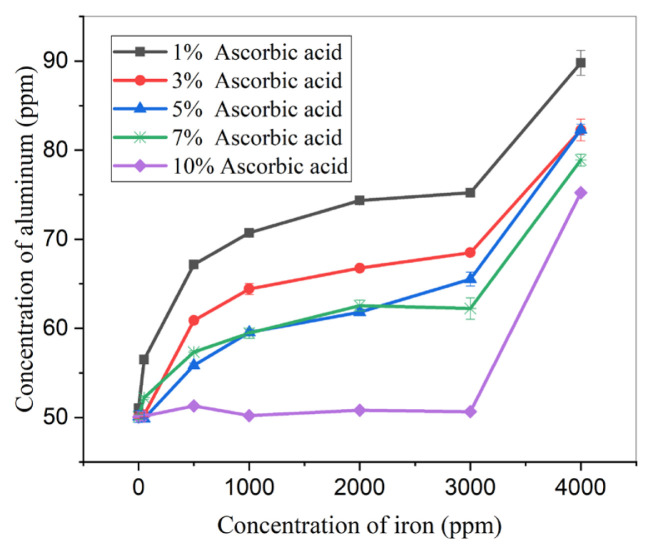
Effect of change in concentration of ascorbic acid and iron on calculated concentration of aluminum (0.14 M CaCl_2_ = 2 mL, buffer solution = 10 mL, pH = 3.6–5.6, 0.05 M ARS = 10 mL, time = 120 min, Al concentration = 50 ppm).

The combined effect of iron and ascorbic acid on the experimentally calculated concentration of aluminum was examined to facilitate the selection of an appropriate concentration of the masking agent for maximum masking. This also shed light on the relationship between the concentration of iron and the corresponding concentration of ascorbic acid needed for effective masking [28]. Figure 3 presents the relationship of ascorbic acid concentration with its masking ability. The iron was respectively masked at up to 0, 50, 100, 500, 1000, 2000, and 3000 ppm by 1%, 3%, 5%, 7%, and 10% ascorbic acid, but the sample with 4000 ppm iron could not be masked by 10% ascorbic acid. Overall, a significant increase in masking capacity was observed when 10% ascorbic acid was used. Hence, the masking capacity of ascorbic acid increases with increasing concentration.

The data further suggested that when the iron content exceeded that of aluminum in the sample, higher aluminum concentrations were measured in experiments utilizing 1%, 3%, 5%, and 7% ascorbic acid. This phenomenon was attributed to the formation of an iron complex with ARS [29]. Therefore, positive interference was observed for the samples containing higher concentrations of iron because of the enrichment of color in those samples.

**Figure 4:**
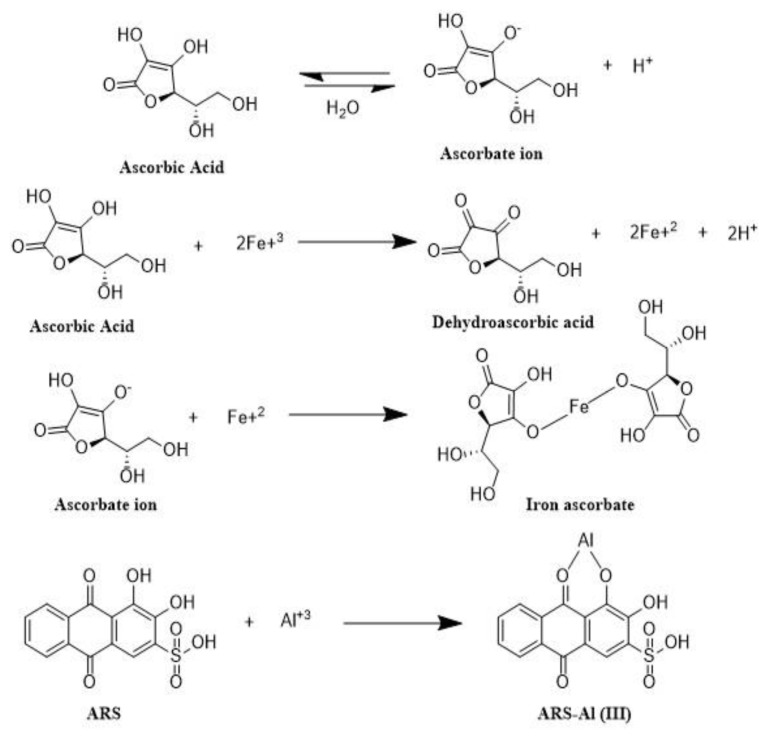
Complex formation of ascorbic acid with iron and ARS with Al(III).

The mechanism behind the masking is the formation of an iron–ascorbate complex. Figure 4 shows the formation of two complexes: ARS–Al(III) and ascorbate–Fe(II). Initially, the ascorbate ion was formed from ascorbic acid, which reduced the ferric ions into ferrous ions. A violet-colored iron–ascorbate complex was then formed as a result of the reaction between ascorbate and ferrous ions. Consequently, total iron was masked completely [30]. When ARS was added in solution, it reacted with aluminum ions and a light orange complex was formed, which exhibited a strong absorption peak at 475 nm [31].

The Al/Fe ratio holds significance as it serves as an indicator of interference occurrence. Table 1 presents the t-test values (calculated t-values, t_cal_) when reference values are known. Each sample was subjected to three measurements and so there were 2 degrees of freedom (*N – 1*). The tabulated t-value (t_tab_) was 4.303 at a 95% confidence limit with 2 degrees of freedom. In accordance with the correct result, this value should be greater than t_cal_. Accordingly, t_tab_ is larger than t_cal_ for samples having Al/Fe ratios equal to or greater than 1.00, 1.00, 1.00, and 0.02 in experiments conducted with 3%, 5%, 7%, and 10% ascorbic acid, respectively. These results are acceptable. Similarly, in the experiments in which 10% ascorbic acid was used as the masking agent, the results of all conducted experiments were adequate as t_tab_ was greater than t_cal_ in all cases except the sample for which 4000 ppm iron was present in the aliquot. Consequently, 10% ascorbic acid effectively masked 3000 ppm iron in aliquots.

#### 3.1.3. Maximum masking limit of 10% ascorbic acid

**Figure 5:**
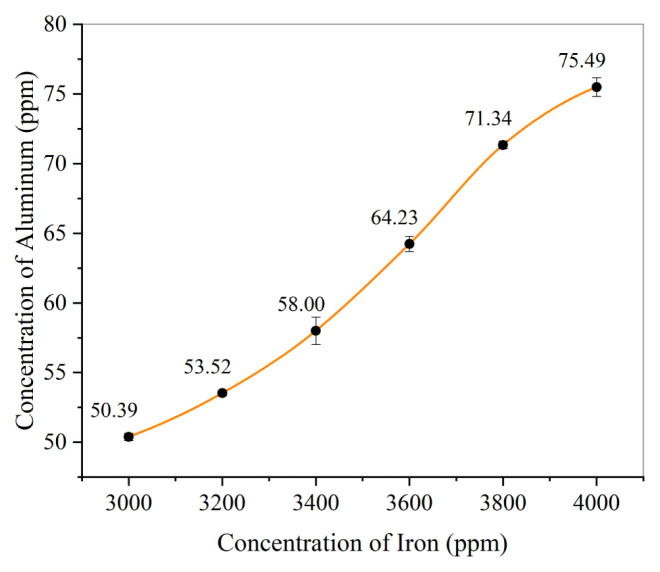
Maximum masking limit of 2 mL of 10% ascorbic acid (0.14 M CaCl_2_ = 2 mL, buffer solution = 10 mL, pH = 3.6–5.6, 0.05 M ARS = 10 mL, time = 120 min, Al concentration = 50 ppm).

The maximum masking limit indicates the quantity of iron that can be effectively masked with 2 mL of 10% ascorbic acid. It is evident in Figure 5 that 2 mL of 10% ascorbic acid effectively masked 3000 ppm of Fe in aliquots, but ascorbic acid did not have this effect at Fe concentrations greater than 3000 ppm. As the concentration of Fe increased, the calculated concentration of aluminum increased due to iron interference. Hence, it was concluded that 2 mL of 10% ascorbic effectively masked Fe at concentrations below 3000 ppm.

#### 3.1.4. Optimization of time

**Figure 6:**
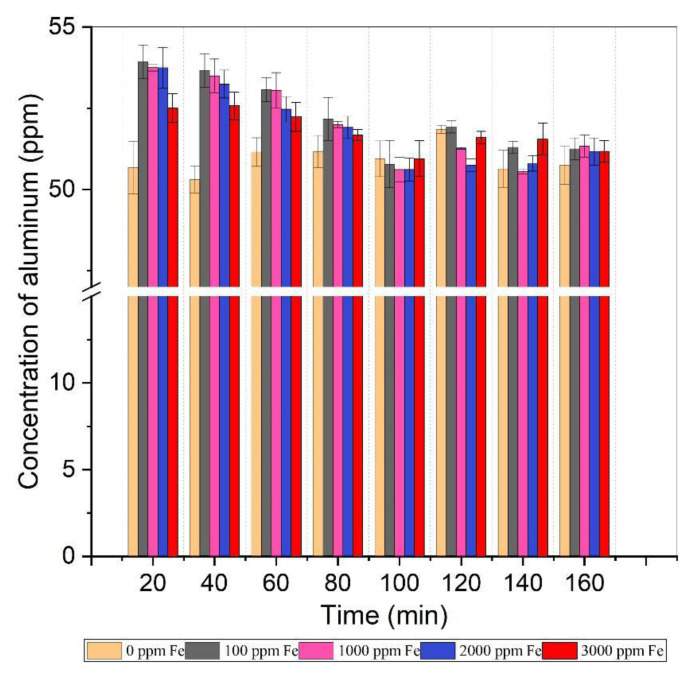
Effect of time variation on determined concentration of aluminum (0.14 M CaCl_2_ = 2 mL, buffer solution = 10 mL, pH = 3.6–5.6, 0.05 M ARS = 10 mL, 10% ascorbic acid = 2 mL, Al concentration = 50 ppm).

Time optimization is important as it provides insight into reaction completion and the time required for complete analysis. For time optimization, eight readings were performed at 20-min time intervals and the concentration of aluminum was monitored. For this experiment, samples prepared as described in Section 2.3.7 were used and the effect of time on the calculated concentration of Al was observed as depicted in Figure 6. Between 20 and 100 min, the calculated concentration of Al exceeded the actual concentration (50 ppm) present in standard samples due to Fe interference. Subsequently, after 100 min, the calculated Al concentration was almost equal to its actual concentration in standard samples. Ascorbic acid reduces ferric ions to ferrous ions, forming a complex with reduced ions. Within the pH range of 3.5–5.6, a ferrous–ascorbate complex formed, although the complete formation of this complex required time. The chemical reaction between ascorbic acid and iron is inherently slow; hence, ARS combined with iron. As the reaction progressed, after 100 min, ascorbate ions reacted with all ferrous ions present in the solution and the complexes of ARS–Al(III) and ascorbate–Fe(II) became stable. In addition, the samples that contained only Al gave accurate concentrations of Al even after 20 min, indicating the reaction time of ARS with aluminum to be 20 min. Fatta et al. reported 10 min as the reaction time for ARS–Al(III) complex formation [32].

The formation of complexes of ARS with iron and aluminum could also be observed through the change in solution color during the course of the reaction. At the start of the reaction, the color of the solution was brownish red for samples with high Fe concentrations. The brownish red color of the solution was due to the formation of the ARS–Fe complex [33]. After 100 min, the color of the solution became light orange. This showed that ascorbate ions had replaced the ARS and all iron present in the sample had been masked by ascorbic acid. The actual color of ARS solution is yellow, but when it complexes with Al(III), it becomes yellowish orange, and when it react with iron, it becomes brownish red. The color of ferrous ascorbate solution is violet around pH 5.6 [34]; therefore, it does not interfere with the reading of the ARS–Al(III) complex at a wavelength 475 nm. Hence, the readings of solutions must be performed after 100 min.

Table 2 contains t_cal_ values when reference values are known. For results to be deemed acceptable, t_tab_ (t_tab_ = 4.303 at 95% confidence limit with 2 degrees of freedom) should be greater than t_cal_. The samples without iron had t_cal_ values greater than t_tab_ even after 20 min. The samples spiked with iron, whose readings were performed from 20 min to less than 80 min, had t_tab_ values lower than t_cal_. Statistically, these results are not acceptable. The samples spiked with iron, whose readings were performed after 100 min, had t_tab_ values greater than t_cal_. Statistically, these results are acceptable. Hence, technically and statistically, it was concluded that the results obtained at 20 min were reliable only for those samples in which no Fe interference was present. Moreover, the results computed after 100 min were reliable for those samples in which iron interference was present.

#### 3.1.5. Effect of change in titanium concentration

The effect of change in concentration of titanium is important as it clarifies the threshold interference concentration of titanium. In spectrophotometric analysis of aluminum, ARS serves as a complexing agent for aluminum; however, ARS also reacts with titanium. The reactivity of ARS with titanium is contingent upon factors such as pH and the concentrations of titanium and ARS.

**Figure 7:**
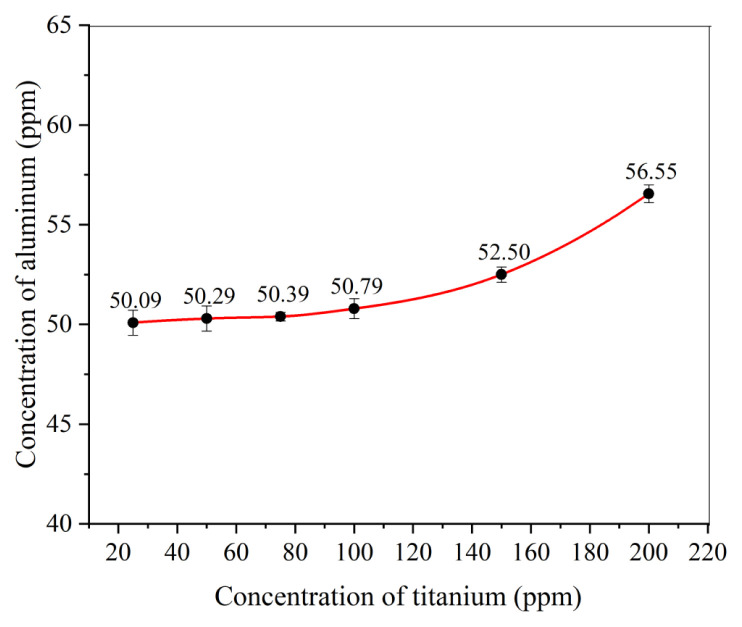
Threshold interference concentration of titanium (0.14 M CaCl_2_ = 2 mL, buffer solution = 10 mL, pH = 3.6–5.6, 0.05 M ARS = 10 mL, 10% ascorbic acid = 2 mL, time = 100 min, Al concentration = 50 ppm).

Figure 7 illustrates the threshold interference concentration of titanium. Titanium exhibited no interference at low concentrations, but interference became evident as its concentration increased. Specifically, interference from titanium was observed when its concentration reached 100 ppm or higher in the aliquot. In the case of solid geological samples, the concentration of titanium in an aliquot is typically less than 100 ppm. Hence, for these samples, titanium does not interfere in the spectrophotometric analysis of aluminum using ARS. However, in some liquid (leached or processed) geological samples, titanium concentrations exceeding 100 ppm were observed in aliquots, resulting in interference. Thus, the threshold interference level of titanium is greater than 100 ppm.

### 3.2. Application of method to soil samples and comparison with results obtained by AAS

The optimized ascorbic acid method was applied to geological samples encompassing various rock types. The concentrations of aluminum calculated with the modified method were compared to those obtained with AAS. Based on the relative concentrations of iron and aluminum, samples were categorized into three types, as also shown in Table 3:

1. Samples 1–4 contained lower concentrations of iron compared to aluminum. In these samples, 2 mL of 10% ascorbic acid masked all of the iron easily. Moreover, the concentrations of aluminum calculated with the modified method and AAS were also comparable.
2. Samples 5–7 had slightly higher concentrations of iron compared to aluminum. In these samples, the concentration of aluminum calculated by the modified method was found to be nearly identical to that obtained through AAS.
3. Samples 8–12 were characterized by significantly higher concentrations of iron compared to aluminum, but ascorbic acid still effectively masked the total iron content in these samples. Furthermore, the results obtained from the ascorbic acid method were similar to those obtained through AAS, indicating comparable outcomes.

Hence, ascorbic acid masked the total iron content in all solid and liquid samples. However, the aliquots of these samples contained titanium at less than 100 ppm.

The precision of the ascorbic acid method as a modified wet chemical procedure, as reflected by the standard deviations of aluminum concentration measurements, shows a moderate level of variability. The standard deviations ranged from ±0.08 to ±0.41, indicating that the repeatability of the method varies depending on the sample and the person performing the experiment. For instance, in Sample 1, the aluminum concentration was 7.14% with a relatively small standard deviation of ±0.08, suggesting good precision in this case. However, in Sample 8, the aluminum concentration was 7.65% with a higher standard deviation of ±0.41, indicating reduced precision. This increased variability in certain samples could be due to personal error or other external factors.

In term of accuracy, the modified wet chemical procedure generally provides values close to those obtained by AAS, which serves as the reference method. In most cases, the aluminum concentrations calculated by this method were within a reasonable range of AAS results, showing good accuracy. For example, for Samples 1, 5, and 8, the difference between the two methods was minimal. However, there were instances in which the modified wet chemical method deviated more significantly from AAS, particularly in the case of Sample 12, where the difference was more pronounced. This could be due to the effect of media, as this sample was a processed sample treated with various acids for the leaching of aluminum and other useful products. Overall, the findings suggest that the modified wet chemical procedure is generally accurate.

### 3.3. Conclusion

Aluminum is abundantly distributed in the earth’s crust. It is present in naturally occurring mineral substances and commercially significant compounds. While the determination of aluminum is a crucial aspect of whole-rock analysis, interference from iron in geological samples poses a challenge. To effectively mask iron interference in geological samples, different masking agents including Tiron, thioglycolic acid, and oxalic acid were evaluated in this study. After optimizing parameters including the concentration of ascorbic acid, the concentration of iron in the aliquot, the masking limit, and the reaction time, ascorbic acid was identified as the most suitable masking agent. It was found that 2 mL of 10% ascorbic acid could mask iron at concentrations of less than 3000 ppm in aliquots. With this masking method, most samples can be easily analyzed for aluminum. Using ascorbic acid as a masking agent in the spectrophotometric analysis of aluminum, particularly when using ARS, enhances the accuracy, safety, and environmental sustainability of the process while reducing costs and ensuring consistent and reliable results. This improvement is crucial for environmental and geochemical studies where precise measurements are essential. Ascorbic acid helps in stabilizing absorbance readings, reducing errors caused by the presence of Fe^+3^ ions that can otherwise distort the results. Therefore, this method produces dependable and reproducible results in spectrophotometric analyses, supporting more accurate environmental assessments and resource management strategies.

## Figures and Tables

**Table 1:** Effect of variation in concentrations of iron and ascorbic acid on calculated concentration of aluminum.

Samples	Percentage of Ascorbic Acid																			
	1%				3%				5%				7%				10%			
	Al Conc. (ppm)	CL*	Al/Fe	T-Test**	Al Conc. (ppm)	CL*	Al/Fe	T-Test**	Al Conc. (ppm)	CL*	Al/Fe	T-Test**	Al Conc. (ppm)	CL*	Al/Fe	T-Test**	Al Conc. (ppm)	CL*	Al/Fe	T-Test**
50–0	51.01 ± 0.53	1.31	-	3.32	50.13 ± 0.23	0.58	-	0.97	50.10 ± 0.65	1.62	-	0.26	50.07 ± 0.27	0.66	-	0.45	50.04 ± 0.16	0.39	-	0.48
50–50	56.49± 0.36	0.89	1	31.38	50.29 ± 0.23	0.58	1	2.18	49.88 ± 0.65	0.16	1	3.13	52.22 ± 0.19	0.47	1	20.48	50.15 ± 0.20	0.49	1	1.34
50–100	61.63 ± 0.12	0.30	0.50	164.73	54.81 ± 0.41	1.02	0.50	20.39	50.26 ± 0.37	0.92	0.50	1.22	53.82 ± 0.13	0.33	0.50	50.40	49.66 ± 0.54	0.44	0.50	-3.29
50–500	67.16 ± 0.37	0.92	0.10	80.22	60.89 ± 0.31	0.78	0.10	60.14	55.85 ± 0.26	0.65	0.10	38.89	57.36 ± 0.16	0.39	0.10	82.02	51.30 ± 1.08	2.69	0.10	2.08
50–1000	70.73 ± 0.40	0.99	0.05	89.86	64.42 ± 0.62	1.55	0.05	40.09	59.56 ± 0.29	0.71	0.05	57.71	59.46 ± 0.52	1.44	0.05	28.11	50.21 ± 0.19	0.47	0.05	1.91
50–2000	74.34 ± 0.30	0.74	0.03	142.04	66.76 ± 0.16	0.40	0.03	181.52	61.82 ± 0.35	0.88	0.03	57.93	62.56 ± 0.59	1.49	0.03	36.39	50.81 ± 0.54	1.35	0.03	2.59
50–3000	75.24 ± 0.32	0.79	0.02	138.12	68.50 ± 0.32	0.80	0.02	100.19	65.53 ± 0.78	1.94	0.02	34.46	62.23 ± 1.21	3.00	0.02	17.56	50.64 ± 0.55	1.37	0.02	2.02
50–4000	89.81 ± 0.31	0.78	0.01	219.25	82.27 ± 0.52	1.30	0.01	107.22	82.32 ± 0.76	1.91	0.01	73.44	78.90 ± 0.31	0.78	0.01	159.21	75.23 ± 0.78	1.92	0.01	56.31

Values are mean (± SD) of three samples analyzed individually in triplicate.

* Confidence interval at confidence limit = 95 % & degree of freedom = 2

** T-Test, when accepted value is known at confidence limit = 95% & degree of freedom = 2

**Table 2:** Effect of time variation on calculated concentration of aluminum.

Samples	Effect of Stay Time Variation on Determined Al Concentration											
	20–25 (min)			40–45 (min)			60–65 (min)			80–85 (min)		
	Al Conc. (ppm)	CL *	T-Test**	Al Conc. (ppm)	CL *	T-Test**	Al Conc. (ppm)	CL *	T-Test**	Al Conc. (ppm)	CL *	T-Test**
50–0	50.68 ± 0.81	2.02	1.45	50.31 ± 0.41	1.03	1.31	51.16 ± 0.44	1.08	4.59	51.17 ± 0.49	1.21	4.19
50–100	52.51 ± 0.51	1.26	8.55	52.58 ± 0.51	1.27	8.76	52.24 ± 0.37	0.92	10.47	51.68 ± 0.66	1.64	4.39
50–1000	53.93 ± 0.62	1.53	11.05	53.67 ± 0.43	1.08	14.63	53.07 ± 0.39	0.96	13.84	52.17 ± 0.34	0.84	11.19
50–3000	53.75 ± 0.11	0.28	57.75	53.25 ± 0.52	1.28	10.93	52.47 ± 0.54	1.35	7.90	51.92 ± 0.09	0.21	39.15
50–3000–2	53.76 ± 0.45	1.11	14.59	53.49 ± 0.43	1.08	14.00	53.06 ± 0.45	1.12	11.80	51.99 ± 0.17	0.41	20.75
**Samples**	**100–105 (min)**			**120–125 (min)**			**140–145 (min)**			**160–165 (min)**		
	**Al Conc. (ppm)**	**CL** *	**T-Test** **	**Al Conc. (ppm)**	**CL** *	**T-Test** **	**Al Conc. (ppm)**	**CL** *	**T-Test** **	**Al Conc. (ppm)**	**CL** *	**T-Test** **
50–0	50.95 ± 0.55	1.37	0.03	51.85 ± 0.12	0.29	5.83	50.64 ± 0.57	1.42	9.52	50.76 ± 0.59	1.46	2.28
50–100	50.95 ± 0.72	1.78	0.03	51.59 ± 0.19	0.49	3.86	51.56 ± 0.18	0.44	13.58	51.17 ± 0.34	0.84	11.38
50–1000	50.78 ± 0.35	0.87	0.03	51.93 ± 0.20	0.50	9.57	51.30 ± 0.24	0.59	11.23	51.25 ± 0.42	1.04	9.15
50–3000	50.62 ± 0.38	0.95	0.02	50.76 ± 0.02	0.06	3.42	50.81 ± 0.08	0.19	60.51	51.17 ± 0.34	0.84	26.69
50–3000–2	50.62 ± 0.55	1.37	0.02	51.26 ± 0.19	0.49	3.98	50.56 ± 0.49	1.22	4.94	51.34 ± 0.33	0.81	4.73

Values are mean (± SD) of three samples analyzed individually in triplicate.

* Confidence interval at confidence limit = 95 % & degree of freedom = 2

** T-Test, when accepted value is known at confidence limit = 95% & degree of freedom = 2

**Table 3:** Comparison of aluminum concentrations calculated by ascorbic acid method and AAS.

Sample No.	Conc. of Ti	Conc. of Fe	Conc. of Al Calculated by Ascorbic Acid Method	Conc. of Al Calculated by AAS
**1**	0.41% ± 0.09	2.46% ± 0.05	7.14% ± 0.08	6.91% ± 0.01
**2**	0.21% ± 0.02	1.12% ± 0.03	6.50% ± 0.28	6.68% ± 0.02
**3**	0.58% ± 0.06	2.17% ± 0.02	5.68% ± 0.17	5.42% ± 0.04
**4**	0.24% ± 0.02	2.16% ± 0.05	7.05% ± 0.18	6.96% ± 0.02
**5**	0.43% ± 0.03	10.94% ± 0.06	6.59% ± 0.12	6.52% ± 0.05
**6**	1.39% ± 0.32	10.94% ± 0.04	6.52% ± 0.28	6.67% ± 0.06
**7**	1.35% ± 0.11	13.16% ± 0.01	6.41% ± 0.26	6.34% ± 0.03
**8**	0.46% ± 0.02	28.79% ± 0.03	7.65% ± 0.41	7.43% ± 0.02
**9**	0.27% ± 0.02	24.86% ± 0.02	5.37% ± 0.32	5.21% ± 0.04
**10**	0.61% ± 0.06	12.37% ± 0.03	0.51% ± 0.40	0.56% ± 0.01
**11**	0.57% ± 0.04	14.29% ± 0.04	1.01% ± 0.35	0.97% ± 0.01
**12**	0.31 g/L ± 0.10	0.33 g/L ± 0.01	0.03 g/L ± 0.01	0.02 g/L ± 0.01

Values are mean (± SD) of three samples analyzed individually in triplicate
